# Bulbil initiation: a comprehensive review on resources, development, and utilisation, with emphasis on molecular mechanisms, advanced technologies, and future prospects

**DOI:** 10.3389/fpls.2024.1343222

**Published:** 2024-04-08

**Authors:** Fuxing Shu, Dongdong Wang, Surendra Sarsaiya, Leilei Jin, Kai Liu, Mengru Zhao, Xin Wang, Zhaoxu Yao, Guoguang Chen, Jishuang Chen

**Affiliations:** ^1^ School of Pharmaceutical Sciences, Nanjing Tech University, Nanjing, Jiangsu, China; ^2^ Bioresource Institute for Healthy Utilization, Zunyi Medical University, Zunyi, Guizhou, China; ^3^ School of Biotechnology and Pharmaceutical Engineering, Nanjing Tech University, Nanjing, Jiangsu, China; ^4^ Bozhou Xinghe Agricultural Development Co., Ltd., Bozhou, Anhui, China; ^5^ Joint Research Center for Chinese Herbal Medicine of Anhui of Institution of Health and Medicine, Bozhou, Anhui Provence, China

**Keywords:** bulbil, bioreactor, terahertz, molecular mechanisms, plant growth regulation, tissue culture, asexual reproduction

## Abstract

Bulbil is an important asexual reproductive structure of bulbil plants. It mainly grows in leaf axils, leaf forks, tubers and the upper and near ground ends of flower stems of plants. They play a significant role in the reproduction of numerous herbaceous plant species by serving as agents of plant propagation, energy reserves, and survival mechanisms in adverse environmental conditions. Despite extensive research on bulbil-plants regarding their resources, development mechanisms, and utilisation, a comprehensive review of bulbil is lacking, hindering progress in exploiting bulbil resources. This paper provides a systematic overview of bulbil research, including bulbil-plant resources, identification of development stages and maturity of bulbils, cellular and molecular mechanisms of bulbil development, factors influencing bulbil development, gene research related to bulbil development, multi-bulbil phenomenon and its significance, medicinal value of bulbils, breeding value of bulbils, and the application of plant tissue culture technology in bulbil production. The application value of the Temporary Immersion Bioreactor System (TIBS) and Terahertz (THz) in bulbil breeding is also discussed, offering a comprehensive blueprint for further bulbil resource development. Additionally, additive, seven areas that require attention are proposed: (1) Utilization of modern network technologies, such as plant recognition apps or websites, to collect and identify bulbous plant resources efficiently and extensively; (2) Further research on cell and tissue structures that influence bulb cell development; (3) Investigation of the network regulatory relationship between genes, proteins, metabolites, and epigenetics in bulbil development; (4) Exploration of the potential utilization value of multiple sprouts, including medicinal, ecological, and horticultural applications; (5) Innovation and optimization of the plant tissue culture system for bulbils; (6) Comprehensive application research of TIBS for large-scale expansion of bulbil production; (7) To find out the common share genetics between bulbils and flowers.

## 1 Introduction

Bulbils, these distinctive plant structures, fulfil a dual role as both agents of reproduction and sources of nourishment (Walck et al., 2010). The development of bulbil is a finely orchestrated process under the influence of various hormones and growth factors, including growth hormone, Cytokinin, Gibberellin, and others (Li et al., 2021a; Wu et al., 2021; Prasad, 2022). These physiological processes involve cell division, differentiation, and morphogenesis, all working in concert to bring about bulbil initiation (Goryachev and Mallo, 2020; Li et al., 2022). Bulbils exhibit a range of shapes and sizes, with variations across plant species, typically adopting spherical, oval, or fusiform forms (Arizaga and Ezcurra, 1995; Xu et al., 2020; Guo et al., 2021). Their exterior presents a characteristic smooth texture, with colours that vary according to the plant species and developmental stage, often displaying shades of green, red, or brown. Internally, bulbil possess a complex structure comprising various tissues, with the cortex and pith playing crucial roles in bulbil development (Luo et al., 2014).

Bulbil, compact, and globular in nature, they typically emerge in specific parts of plants, such as leaf axils, stems, or inflorescences, primarily serving as a means of asexual reproduction (She et al., 2018; He et al., 2020). They remain relatively immobile and can be detached from the parent plant upon maturity, allowing them to take root and develop into new plants (Wang J. et al., 2023). These bulbil act as biological clones, originating from the mitotic division of a single parent cell and thus inheriting all the genetic information at the initiation of the parent plant (Abraham et al., 2015). Terahertz waves, commonly referred to as THz waves or terahertz radiation, occupy a unique electromagnetic spectrum situated between microwaves and infrared light, typically spanning a frequency range from 0.1 to 10 THz (Li et al., 2021b). Recent advancements in technology have unveiled the significant potential of terahertz technology across a broad spectrum of applications (Zahid et al., 2019; Yang et al., 2020). While it has found substantial use in medical treatments and communication equipment, recent research has provided evidence suggesting that THz waves can stimulate plant growth. Additionally, a growing body of evidence points to the potential role of THz waves in regulating the production of plant bulbil (Wang D. et al., 2023).

**Figure 1:**
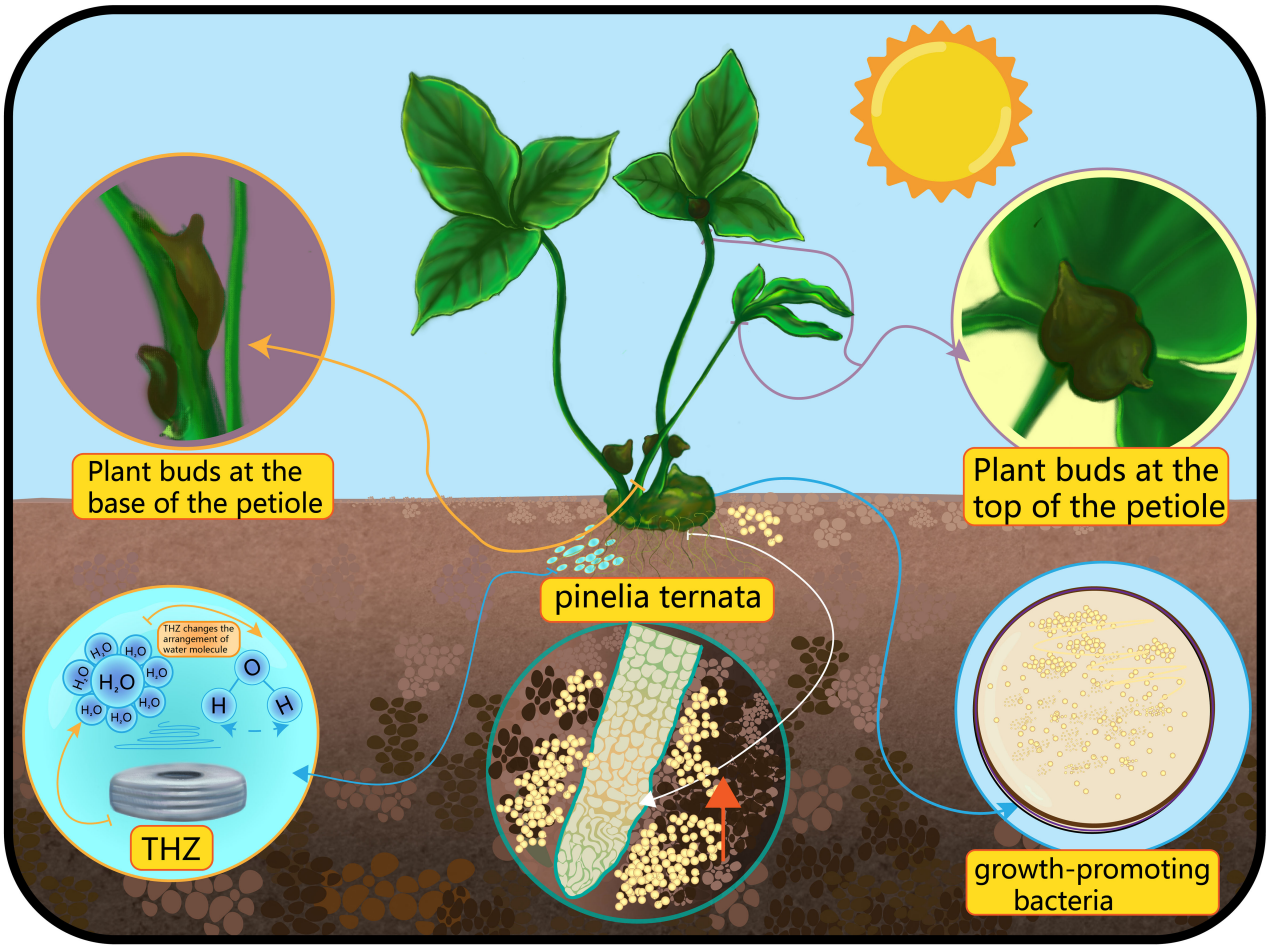
The picture shows a schematic diagram of the interaction between THZ, microorganisms, moisture, and sunlight factors on the formation of bulbils at the bottom and top of *Pinellia ternata*.

In recent years, the realm of plant biology research has evolved significantly, with emerging technologies and innovative approaches shedding new light on bulbil initiation. One such advancement involves the exploration of terahertz (THz) frequency band interactions with plants, unveiling previously uncharted dimensions of plant adaptation and regulation in response to complex growth environments (Wang D. et al., 2023). This review article delves into the multifaceted realm of bulbil initiation, offering a comprehensive analysis of the mechanisms that govern their production and the regulatory factors that underpin this intriguing process. Through studying the molecular foundations, gene clusters, and practical uses of bulbil production in certain plant species, we hope to gain a better understanding of how plants reproduce and their amazing ability to adapt to different environmental challenges. The insights gleaned from this exploration have significant practical implications, spanning the fields of ecology, horticulture, and medicine, and offer valuable knowledge for enhancing crop cultivation techniques, advancing drug development, and promoting ecosystem conservation (see **Figure 1**).

## 2 Plants with bulbils and characteristics of their bulbils

**Figure 2:**
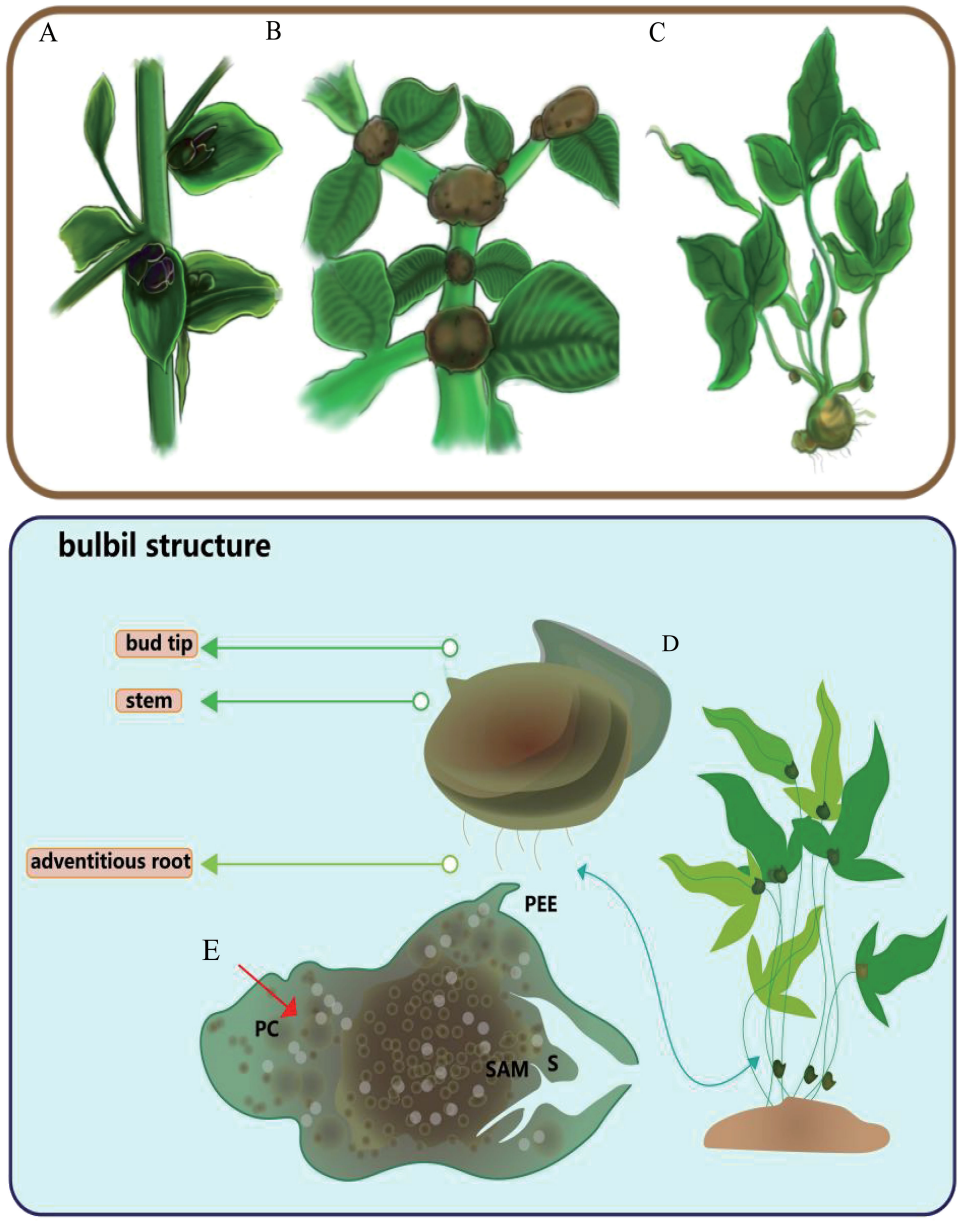
Three kinds of typical bulbil-plants, typical bulbil position and structure. **(A)** represents the common positions of *Lilium* bulbils, **(B)** represents the common positions of *Amorphophallus muelleri* bulbils, and **(C)** shows the common positions of *Pinellia ternata* bulbils. **(D)** represents the appearance structure of the *pinellia ternata* bulbil. **(E)** represents the anatomical structures of *pinellia ternata* bulbil, PC for parenchyma cells, S for bud scales, PEE for epidermis-derived structures, and SAM for the apical meristem.

**Figure 3:**
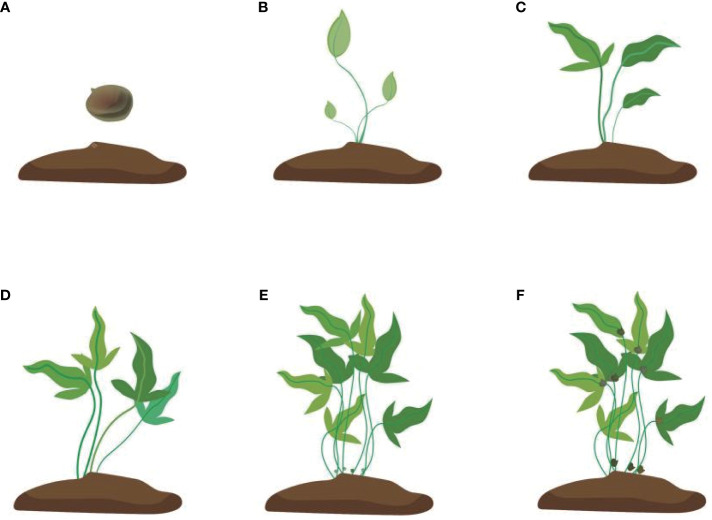
Development of the bulbil of *Pinellia ternata*. **(A)** stands for tubers just planted in the soil, **(B)** stands for seedlings just growing, and **(C)** stands for seedlings whose leaves are fully expanded. At this time, the bulbil generally starts to initiate but cannot be seen; **(D)** stands for stronger photosynthesis. At this time, some white spots protrude from the stem, but they are still not easy to see. **(E)** stands for the bulbil starting to expand, and **(F)** stands for the mature colour of the bulbil turning brown.

Plants that exhibit bulbil structures are primarily found in the *Araceae* family, specifically in the genera *Pinellia* and *Amorphophallus*, as well as in the genera *Lilium* in the *Liliaceae* family, *Allium* in the *Amaryllidaceae* family, *Dioscorea* in the *Dioscoreaceae* family, *Polygonum* in the *Polygonaceae* family, *Titanotrichum oldhamii (Hemsl.) Soler.* in the *Gesneriaceae* family, and *Alocasia* in the *Araceae* family, among others, and the appearance and anatomical maps of the pinellia ternata bulbil are provided (see **Figure 2**). The bulbils of these plants are generally spherical or oval, though other irregular shapes also occur, depending on the specific characteristics and physiological state of the plant in the *Araceae* family. They are much smaller than those of the *Amorphophallus* in the same family (Arizaga and Ezcurra, 1995; Guo et al., 2021). Regarding colour, the bulbils of *Pinellia* start off green and eventually turn black or brown as they grow (see **Figure 3**). The position of bulbil attachment varies among different plants and is primarily categorised as follows: (1) at the lower end of the petiole or at the junction of leaflets, as seen in *Pinellia* and *Amorphophallus*; (2) in the leaf axils or at the stem nodes, as observed in *Lilium*, *Linum usitatissimum L.*, and *Dioscorea opposita*; (3) on the flower stalk or in the inflorescence, as in *Allium*, *Titanotrichum oldhamii* (Hemsl.) Soler., and *Agave americana L.* (see **Table 1**).

**Table 1:** Common Bulbils plants reported in the literature.

Genera	species
*Lilium L.*	*Lilium lancifolium*
	*Lilium bulbiferum)*
	*Lilium sargentiae*
	*Lilium sulphureum*
*Dioscorea L.*	*Dioscorea batatas*
	*Dioscorea aculeata*
	*Dioscorea alata*
	*Dioscorea bulbifera*
	*Dioscorea kamoonensis*
	*Dioscorea opposita Thunb.*
	*Dioscorea pentaphylla*
*Pinellia*	*Pinellia ternata*
	*Pinellia pedatisecta Schott*
	*Pinellia cordata*
*Amorphophallus*	*Amorphophallus bulbifer*
	*Amorphophallus konjac*
	*Amorphophallus muelleri Blume*
	*Amorphophallus albus*
	*Maitake Amorphophallus konjac*
*Polygonum L.*	*Pinellia integrifolia N. E. Brown*
*Allium*	*Allium sativum*
	*Allium cepa L.*
*Agave*	*Agave macroacantha*
	*Agave tequilana*
	*Agave americana L*
*Titanotrichum*	*Titanotrichum oldhamii*
*Sedum*	*Sedum telephium*
	*Sedum bulbiferum*
*Sagittaria*	*Sagittaria wuyiensis*
*Lycoris*	*Lycoris radiata*
*Elatostema*	*Elatostema involucratum*
*Bistortaviv*	*Bistortavivipara(L.) Gray*
*Polygonum*	*Polygonum viviparum L*
*Curculigo*	*Curculigo orchioides*
*Laportea*	*Laportea bulbifera*

## 3 Development of bulbils

### 3.1 Cellular and tissue basis of bulbil development

The development stages of plant bulbils generally include initiation, growth, differentiation, and maturation (Luo et al., 2014; She et al., 2018). Different plants exhibit varying developmental pathways for their bulbils. For example, in the *Lilium lancifolium* Ker Gawl. (a type of plant used for flowers, food, and medicine), bulbils morphologically progress from ‘torpedo shape’ to ‘embryo shape’ and finally to ‘spherical shape’ during their development (Zhang, 2017). Anatomical studies reveal that *Lilium lancifolium* Ker Gawl. bulbils are composed of scales, bulbils, and adventitious roots (Fan et al., 2019). The outer epidermal cells of scales have a developed cuticle, and the 1-2 layers of mesophyll cells beneath the epidermis contain anthocyanins; these mesophyll cells are filled with green chloroplasts. The green chloroplasts at the base of the first to third scales are concentrated in the direction where the adventitious roots elongate. The pigment content in scales decreases from the outside inward; the scales’ vascular bundles are composed of phloem outside. The bulbil primarily consists of a cortex and vascular column. The upper end of the bulbil includes the apical meristem and bulbil sheath, while cell death occurs at the lower end, although no abscission layer similar to that found at the base of the petiole during leaf fall is observed. The adventitious roots originate from the pericycle cells of the second scale and are separated from the surrounding scale tissue (Zhu, 2018). In the *Araceae* family, plants in the *Pinellia* genus, such as *Pinellia ternata* and *Pinellia cordata N. E. Br.*, can be divided into three visible stages: no apparent phenomenon, early protruding bulbil structure, and swelling to maturity. Anatomically, the development of the bulbil is divided into three stages: initiation of the bulbil primordium, differentiation of the bulbil, and swelling to maturity. The initiation cells of the bulbil originate from the subepidermal parenchyma cells on the ventral side of the petiole. In the later stages of primordium development, new growth points and scale leaves are differentiated, and the mature bulbil’s inner parenchyma cells contain a large amount of nutrients (Luo et al., 2014). In the *Amorphophallus* genus, including the *Maitreya Amorphophallus*, bulbils grow at the fork of the petiole and on the split leaves. They come from cells under the skin of the main stem, or petiole, that have the ability to divide again (She et al., 2018). The bulbil grows in three stages, which are similar to those of the *Pinellia* genus: the bulbil primordium initiates to form, the bulbil gets bigger, and the bulbil matures (Luo et al., 2014). It is evident that the cellular and tissue development of bulbils in different plants has certain differences among species, but the origin of bulbil development is always the meristematic cells at the site of bulbil attachment (Xu et al., 2020; Li et al., 2022).

### 3.2 Determination of bulbil maturity

The maturation of bulbils is the final stage of their development, and determining their maturity is crucial for harvesting, preservation, and utilization. According to literature, the development of *Pinellia ternata* bulbils ceases with the ageing and toppling of the plant, unlike the clear maturation stage seen in *Lilium lancifolium Thunb.* Therefore, the development of *Pinellia ternata* bulbils can be roughly divided into the initiation of the bulbil primordium, the differentiation stage, and the swelling stage, without a distinct maturation stage (Luo et al., 2014; Fan et al., 2022). However, this seems to be a common phenomenon in all plants; bulbils do not develop after the plant topples, including in plants like *Lilium lancifolium Thunb* (Zhang et al., 2022; Wang D. et al., 2023) and *Dioscorea oppositas* (Li et al., 2023b). In fact, the toppling of *Pinellia ternata* is not a mandatory periodic behaviour but rather a response to drought stress, indicating that *Pinellia ternata* also has a distinct maturation stage. The effect of drought on the development of plants will be explained in the following sections. So, how can we determine whether a bulbil is mature? The amount of sugar and dry matter in the bulbils of *Dioscorea opposita* and *Dioscorea bulbifera L.* changed over the course of their development (Long et al., 2011, 2013). It was discovered that the sugar and dry matter in the bulbils only fully accumulate after the stems die and the bulbils turn brown. In different strains of *Pinellia ternata*, the bulbils show significant colour variations at maturity, mainly turning brown or dark black (Luo et al., 2014). This indicates that the morphology and colour of bulbils can be used to determine their maturity.

## 4 Factors affecting the development of bulbils

### 4.1 Effects of phytohormones on bulbils initiation

Phytohormones, also known as plant hormones or growth regulators, play a pivotal role in the growth and development of plants. They are involved in various physiological processes, such as cell division, elongation, differentiation, and responses to environmental stimulation. Plant hormones play an important role in the development of bulbil. We explored the effects of these hormones in more detail.

#### 4.1.1 Auxin/IAA

Studies based on transcriptomic data during the initiation of bulbils suggest that IAA may promote the initiation of bulbils but inhibit the further initiation of bulbils, and the conclusions drawn from hormone content measurements targeting each developmental stage are consistent with the transcriptomic data (Yang et al., 2017; Fan et al., 2019b). In *Pinellia ternata*, IAA delivered through leaves promotes the early development of bulbils, while IAA originating from leaves decreases during the expansion stage, and the gradual increase of autonomously synthesised IAA promotes further expansion of *Pinellia ternata* bulbils. IAA helps to promote the initiation of plant bulbils (Xia, 2019). In *Agave americana L.*, AtqPIN1 and AtqSoPIN1 have the ability to coordinate IAA transport to promote the initiation of bulbils (Abraham et al., 2015). During bulbil development, auxin depletion is slowly consumed along the stem, so that the bulbil bulbils in the upper part of the stem will be affected more than those in the lower part of the stem. However, auxin application to remove apical wounds did not completely inhibit induced bulbil bulbilding, as did bulbil growth in vegetable beans and Arabidopsis (Nonhebel, 2015). In the Asiatic hybrid lily, IAA does not act alone. Its ratio to zeatin (a cytokinin) suggests that IAA may cooperate with ZT; the content of IAA showed a significant negative correlation with the bulblet initiation rate (Liang et al., 2023). In *Lilium oriental* hybrid ‘Sorbonne’, IAA application could induce the generation of bulbils (Li et al., 2019). After removing the tip, IAA was able to induce bulbil initiation around the top wound, a process similar to inducing the callus (Li et al., 2022). However, In *Lilium lancifolium*, IAA may have a dual effect on the initiation of bulbils, first promoting their initiation and subsequently inhibiting their growth (Yang et al., 2017). In *Lycoris radiata*, zeatin (ZT) is a kind of natural cytokinins, the ratio of ZT/IAA is a key factor in inducing bulbil initiation (Xu et al., 2020). *Agave tequilana* showed that IAA inhibited bulbil development (Abraham et al., 2015). In *Lycoris radiata*, the content of endogenous IAA tended to increase and decrease during bulbil initiation and development, respectively (Xu et al., 2020). Studies in *Dioscorea opposita* also showed that a temporary increase in local Auxin/IAA promoted bulbil initiation, after which further depletion or export from bulbil was observed (Li et al., 2020). From this, it is hypothesised that growth hormone acts at the initiation stage of the plant bulbil and is secondary to subsequent development. Although, Auxin/IAA plays a role in bulbil development, its direct or indirect role is the focus of researchers. The bulbil initiation in lilies is similar to the initiation of axillary bulbils (AXBs) (Li et al., 2014; Wang and Jiao, 2018). During the initiation of AXBs, IAA exhibits an indirect effect, namely being present in the stem rather than entering into the AXBs (Muller and Leyser, 2011). Another study showed that AXB growth induced by head and cytokinins (CKs) was independent of auxin flux in the bulbil at the early stage (Chabikwa et al., 2019).Therefore, auxin may regulate branching indirectly by regulating other hormones, such as CKs and strigolactones (SL) (Muller and Leyser, 2011; Chabikwa et al., 2019; Luo et al., 2021a). It has also been suggested that SL plays an inhibitory role for bulbil in downstream of auxin (Li et al., 2019).

In addition, some auxin analogues such as NAA, indole propionic acid, indole-3-butyric acid (IBA), 2,4-D, 4-iodophenoxyacetic acid, etc. can all play the role of auxin and may play an important role in the regulation of bulbil initiation or development. In *Amorphophallus muelleri Blume*, the use of NAA enables the bulbils to promote the growth of different specifications, which tends to accelerate the emergence time of new bulbils (Yoseva et al., 2022). But not in all plants; NAA promotes bulbil development. For example, in *Hippeastrum vittatum (Amaryllidaceae)*, the application of exogenous auxin does not result in scale propagation or bulbil development but significantly increases the number of rotting scales (Zhang et al., 2013). During plant tissue culture, the common foreign auxin analogues NAA and 2,4-D are often used in plant tissue culture to induce callus (Tanida and Shiota, 2019; Liu et al., 2023). As mentioned earlier, the process of bulbil initiation around the wound is similar to the process of callus initiation in lilies (Li et al., 2022). In *Hyacinthus orientalis*, the use of IBA also had positive effects on bulbil proliferation (Miyamoto et al., 2015). Overall, IAA plays a dual regulatory role in bulbil initiation and later development in different plants, and the bulbil-induced effect of exogenous auxin analogues may vary for different plants.

#### 4.1.2 Brassinolide and cytokinins

**Figure 4:**
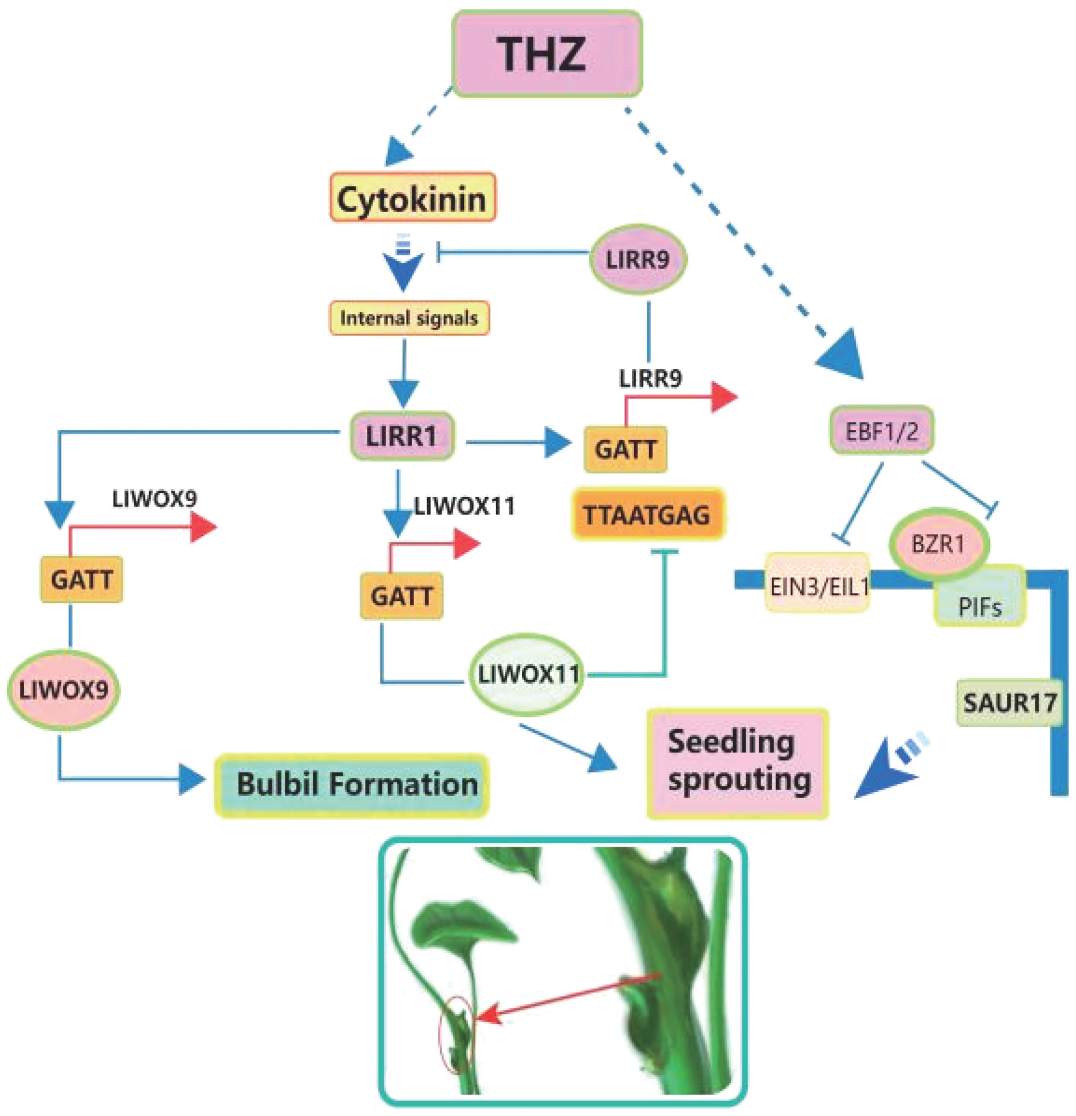
Intention of cytokinin on *Pinellia ternata* to produce bulbils.

Brassinolide (BR) and cytokinins (CK) are two important groups of compounds that control plant growth. They play important roles in many areas of plant biology, especially in asexual reproduction processes like the creation of bulbils (Harashima and Schnittger, 2010; Wu et al., 2021). Both BR and cytokinins are recognised as key plant growth regulators actively involved in steering the growth and development of plants, with a particular focus on their influence in the intricate process of bulbil initiation (see **Figure 4**).

**Figure 5:**
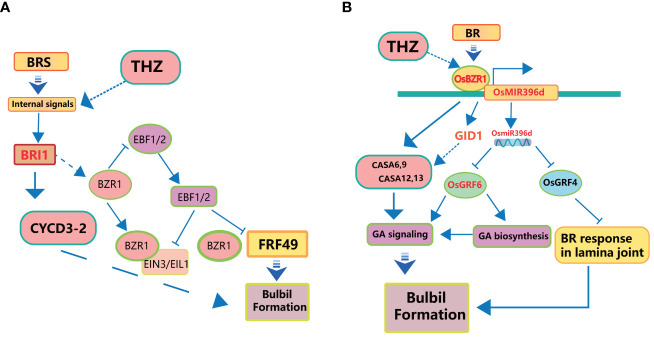
Schematic diagram of the interaction mechanism of BR on *Pinellia ternata* bulbil. **(A)** represents THZ and BR promoting bulbil formation through CYCD and ARF. **(B)** represents BR promoting bulbil formation through GA signaling and BR response.

BR, a class of naturally occurring phytohormones, plays a central role in several fundamental plant processes, including cell elongation, cell division, phototropism, gravitropic responses, and so on (Otie et al., 2021). In the context of bulbil initiation, BR assumes multiple crucial functions (**Figure 5A**). BR can promote photosynthesis in plants and increase the assimilation rate of leaf carbon (Hu et al., 2013).

Firstly, it facilitates cell elongation by relaxing the cell wall (Hoson et al., 1991), rendering it more pliable and thus promoting easier cell elongation. Additionally, BR stimulates cells to enter the cell cycle, encouraging cell division (Zhiponova et al., 2013). Furthermore, BR exerts influence on gene expression by interacting with specific proteins, either activating or inhibiting the expression of genes associated with growth and differentiation (Guo et al., 2021). Lastly, BR’s signalling pathways often involve interactions with AUX/IAA (Auxin/Indole-3-Acetic Acid) proteins and ARF (Auxin Response Factor) proteins (Guo et al., 2021; Li et al., 2022). Aux/IAA proteins function in auxin and BR signaling pathways, and IAA proteins function as signaling components regulating BR sensitivity in a manner depending on the organ type (Nakamura et al., 2006). An increase in BR concentration triggers the ubiquitination and subsequent degradation of the AUX/IAA protein, releasing ARF (Song et al., 2009). ARF, in turn, can regulate the expression of downstream genes (Wang et al., 2018). These multifaceted roles of BR are integral to understanding its contribution to bulbil initiation (**Figure 5B**).

CK has an important role in bulbil-initiated exogenous and subsequent development (Tanaka et al., 2006). Currently, in plants, there are only two active cytokinins, namely, and isopentenyladenine and its hydroxylated derivative zeatin (ZT) (Letham, 1963; Frebort et al., 2011). In *Lilium lancifolium*, cytokinins (CKs) promote the initiation of bulbil at the leaf axils (He et al., 2020). In *Lycoris chinensis*, and a high ratio of ZT/IAA may initiate bulbil initiation (Prasad, 2022). Recent studies show that division analogues such as: 6-benzylaminopurine (6-BA), a commonly used experimental synthetic cytokinin, induce bulbil initiation in *Lilium lancifolium* by promoting cell proliferation (Mo et al., 2023). N- (2-chloro-4-pyridyl) -N ′ -phenylurea (CPPU) and 2-isopentenyladenine (2-iP) play a role in the initiation of bulbil, but they promote bulbil development in the expression of cytokinin receptor gene and increase the ratio of GA3 and IAA to cytokinin (Prasad, 2022). This implies that the role of CKs has a positive effect on bulbil development in different bulbil plants, but maybe in different plants, Cytokinin may crosstalk with other hormones to promote bulbil development. This provides important insight onto the role of cytokinin in other bulbil development.

#### 4.1.3 Gibberellin

Gibberellin (GA), a growth-promoting hormone, plays a crucial role in shaping plants in *Pinellia ternata* through multifaceted mechanisms (Zhang et al., 2018). GA stimulates stem elongation (Yu et al., 2021), a pivotal process contributing to bulbils initiation. In *Lilium lancifolium Thunb.*, the size of bulbil was also significantly higher than the control treatment when sprayed GA3 at 50 mg/L, but the number of bulbil produced by a single plant was not higher than the control, which implies that GA3 acts in the late stage of bulbil development rather than in the initiation of bulbil (Cai, 2021). Another study proved this. By spraying GA3 and its inhibitor (PAC), GA3 inhibited bulbil initiation, reduced the number of single plants but increased the weight of single bulbil, and the parent bulbil could germinate directly without dormancy; PAC treatment significantly increased the bulbil thickness with the highest differentiation efficiency of 56.27% (Wang J. et al., 2023). It regulates stem elongation by activating growth hormone-responsive genes, such as *GAI* and *RGA*, while also being associated with genes essential for bulbils induction and development, including LsTFL1 (Zhang et al., 2019a). In Asiatic hybrid lilies, there was a significant positive correlation between the accumulation of GA3 and the bulblet initiation rate. The spraying of exogenous GA has a significant effect on plant bulbil development, which may reduce the yield of bulbil on the plant and increase the length of individual bulbil. It was shown that low concentrations of GA3 slightly increased the bulbil yield, while high concentrations of GA3 inhibited the bulbil initiation (Kim et al., 2003). Moreover, exogenous GA treatment will increase the length of yam sprouts and reduce the deformity rate of bulbil, but will significantly inhibit the yield of bulbil on a single plant (Gong et al., 2015). In addition, GA3 treatment also has an impact on the development of bulbils in *Lilium lancifolium Ker Gawl*. Consistent with research in *Dioscorea opposita Thunb.*, GA3 treatment can also increase the length of bulbils (Zhang et al., 2019a). Therefore, GA has dual effects on the initiation and development of bulbil.

#### 4.1.4 Abscisic acid

Abscisic acid (ABA) is characterised as an inhibitory hormone. In higher plants, the *de novo* synthesis pathway involves carotenoids (Nambara and Marion-Poll, 2005). ABA signaling begins when ABA receptors sense ABA signaling and then transmits ABA signals downward through a series of intracellular regulators, forming a complex intracellular signal transduction network that eventually transforms into visible physiological effects (Hauser et al., 2011). ABA is well-known for its important role in seed germination and drought resistance (Liu et al., 2022; Zhang and Bao, 2023). Recently, the role of ABA in bulbil development has been continuously reported, such as when the promotion of ABA content inhibited the bulbil development of *pinellia ternata* (Guo et al., 2021). The decrease in ABA content contributes to bulbil initiation. Primarily, ABA is associated with growth inhibition, typically restraining stem elongation, while also assuming a pivotal role in the dormant phase of bulbil (Guo et al., 2021). ABA also often crosstalk with other hormones during bulbil initiation. For example, the amount of ABA after brassinosteroid (BR) treatment decreases, and when BR blocker is used, the development of *pinellia ternata* bulbil is blocked (Guo et al., 2021).In conclusion, ABA can regulate bulbil development through its regulatory role with other plant hormones.

#### 4.1.5 Other plant hormones

Ethylene, salicylic acid (SA), and jasmonic acid (JA) play important roles in plant defence (Li et al., 2018; Barbier et al., 2019; Luo et al., 2021b). In *Lycoris radiata*, during bulbil initiation, ethylene synthesis-related genes are downregulated, while JA and SA synthesis genes are upregulated. These hormones appear to be involved in the initiation of bulbil, but unfortunately, the content of these three hormones was not determined (Xu et al., 2020). In *Amarine*, elevated JA levels were also associated with bulbil initiation, the underlying mechanism may be that the jasmonate acid (JA) wave cooperates with histone methylation to upregulate a pulse of auxin production (Zhang et al., 2019b), and then promote the initiation of bulbil. Ethylene, SA, and JA are known to promote senescence, whereas some hormones, including cytokinin (CK) and auxin suppress it (Zhao et al., 2017). When plants undergo photosynthesis, rather than the seed state, the increased senescence of leaves in extreme environments stimulates the expression of these hormones, which can induce SAGs to shift nutrients down from the tip (Zhao et al., 2017). While the bulbil is the typical nutrient storage organ of the bulbil plants, the volume of the natural bulbil will increase rapidly. Strigolactones (SLs) are a class of multifunctional plant metabolites that not only play an allelic role in the rhizosphere but are also a new type of hormone that regulates plant growth and development (Yoneyama and Brewer, 2021). During bulbil development, SL may cooperate with auxin to regulate plant branching (Luo et al., 2021a). Branching can increase the number of bulbils, but the mechanism related to bulbil development may be SL and auxin inhibiting bulbil initiation (Leyser, 2009). Pcz treatment significantly increased the SL content in the bulbil, while PCZ treatment inhibited the development of bulbils (Guo et al., 2021), suggesting that SL is a type of hormone that is unfavourable for bulbil development. In summary, these hormones play different roles in the initiation and later development of bulbil, either promoting or inhibiting them. However, current research is still in its early stages, and further research is needed on their specific regulatory networks.

### 4.2 The role of sugars in bulbil initiation and development

In most plants, starch and sucrose are the main forms of carbohydrate transportation and storage, respectively. The accumulation of starch is a dynamic process synthesised by the storage organ (bulbil) from the cracking products of sucrose, including the synthesis, transinitiation, transportation, and degradation of starch and sucrose (Zhang et al., 2017). Earlier studies on single leaf culture of daffodils found that, in the presence of the growth hormone NAA, 176 mM of sucrose strongly promoted the initiation of bulbils (Barrett et al., 2004). Moreover, the process must be sucrose to induce bulbil initiation, not other sugars. In addition, the initiation of bulbils was not entirely dependent on the carbon source and energy supply characteristics of sucrose (Yang et al., 2017). In *Pinellia ternata*, BR enhances the development of bulbils. During this process, the starch decomposition metabolism to maltodextrin and maltose was decreased, and the cellulose decomposition metabolism to D-glucose was increased in bulbils (Guo et al., 2021). Previous studies have shown that soluble sugar, especially sugars, are inducers of underground storage organ initiation (Fernie and Willmitzer, 2001). It is worth noting that both bulbils and tubers are nutrient storage organs, which may also be an important factor in the late development of bulbils. Later studies showed that this point was verified. For example, in *Pinellia ternata*, BR treatment promoted the late development of bulbil, and a large number of starch particles were observed in this process (Goryachev and Mallo, 2020). In *garlic*, sucrose may act as a key signal for the initiation of bulbils (Peng et al., 2021). The production and storage of starch may help the initiation of bulbils on the upper part of *Lilium lancifolium Ker Gawl.* (Yang et al., 2017). As the bulbils develop and mature, the number of starch granules in cells gradually decreases, but their relative area increases, showing that starch accumulation plays an important role in the initiation of *Lilium lancifolium Ker Gawl*. lily bulbils (Yang et al., 2017). In plant tissue culture, high concentrations of sucrose are conducive to the initiation of stem bulbils (Fki et al., 2011). In short, sucroseis essential for bulbil morphogenesis, while starch is essential for bulbil emergence and development.

### 4.3 Environmental factors

Light, temperature, and humidity are the primary environmental factors affecting the initiation of plant bulbils. It is preliminarily speculated that these factors regulate the initiation and development of bulbils mainly by influencing the accumulation of nutrients in plants and the pathways of sexual reproduction.

Light affects the initiation of plant bulbils mainly through light intensity, quality, and photoperiod. In *Pinellia ternata*, strong light can cause the plant to topple, and shading treatment can enhance its yield by regulating its growth state through long non-coding RNAs (Yang et al., 2023a) and DNA methylation (Shi et al., 2020). These processes have a significant impact on bulbil development, and optimal growth conditions provide the basic material foundation for bulbil initiation. When light intensity reaches 8,618 Lux, *Pinellia ternata* bulbils show strong vitality, the highest germination potential, and maximum bulbil length and shoot width. In terms of light quality, red light promotes the initiation and number of *Pinellia ternata* bulbils (Chen et al., 2013). Under low light intensity, *Agave americana L*. produces bulbils at the base of small bracts (Abraham et al., 2015). Consistently, the number of *Dioscorea opposita* bulbils increases (Li et al., 2023b). The impact of photoperiods differs from light quality. In *Dioscorea opposita*, the effect of photoperiod is secondary to light quality. Analysis of the relationship between photoperiod and bulbil growth shows that under various light intensities, 4 h/d of light exposure is insufficient for rapid bulbil growth, while 16 h/d inhibits growth to varying degrees (Li et al., 2023b). However, 8 and 10 h/d of light exposure are suitable for bulbil growth (Bilu et al., 2020). The bulbils of *Laportea bulbifera* and *Elatostema involucratum* form under short-day conditions. while *Allium sativum L.* and *Allium cepa L.* need long-day conditions to form bulbil (Le Guen-Le et al., 2002; Atif et al., 2021). *Dioscorea alata* L. bulbil initiation is unaffected by photoperiod (Okagami and Kawai, 1977), short-day, slightly concealed environments encourage the production of more bulbils in *Titanotrichum oldhamii (Hemsl.) Soler.*, whereas long-day, full-light environments inhibit bulbil’s production (Le Guen-Le et al., 2002). Note that light affects sugar metabolism, especially starch accumulation, and previous descriptions have shown that sugar metabolism plays an important role in the development of the bulbil.

Humid environments are generally cooler and shadier. *Pinellia ternata*, *Laportea bulbifera* and *Amorphophallus konjac* grow in relatively dark, super-moist habitats. It is speculated that these habitats hinder plant pollination, reduce seed production, suppress sexual reproduction, and thus lead to the evolution of bulbil traits, ensuring species continuity through asexual reproduction. Moreover, this integrated optimal humidity environment promotes maximum photosynthesis, leading to more storage of sugars, which in turn promotes the later development of bulbil bulbil. Generally, acute water shortages lead to *Pinellia ternata* sprout tumbles (Huang et al., 2019). Drought will lead to an increase in ABA content (Xue et al., 2007) and a large number of ROS. The purpose of ABA promotion is to clarify the ROS level upregulated by drought, and the most direct evidence is that the exogenous use of ABA can reduce the accumulation of ROS (Dong et al., 2021). However, ROS is a small signal molecule of IAA and CK. IAA and CK (Tognetti et al., 2017) can induce ROS production, and ROS in turn binds with IAA to form IAA ox to rejoin the hormone circulation (Dong et al., 2021). It has been said that an increase in ABA content will inhibit the development of bulbils. And this process happens exactly the reduction of ROS. However, this inhibition of bulbil development is useful in the early stages of plant growth. Under sudden drought stress, plants will rapidly transfer nutrients from the leaves and petioles to the bulbils, causing rapid bulbil development during this time. This process is closely related to ethylene because ethylene induces the senescence of leaves and other tissues under drought conditions and induces nutrient transfer (Luo et al., 2021b). The high salt environment will also lead to the rise of ABA and the rise of ROS. When ABA rises to a certain extent, ROS is removed and the content decreases (Meng et al., 2020).

Lower temperatures slow down plant growth and development. For example, with increasing altitude on the Tibetan Plateau, the inflorescence height of *Bistorta vivipara* (L.) Gray decreases, as do the number of flowers and bulbils (Fan and Yang, 2009). This indicates that the low temperatures in high-altitude areas affect bulbil development. The shortening of inflorescences due to low temperatures does not provide an ecological niche for bulbil initiation, thus significantly reducing their number. It is noteworthy that low temperatures affect the efficiency of photosynthesis (Zhang et al., 2019b); this means that the reduction of sugars is unfavourable to the development of bulbils.

## 5 Gene regulatory networks associated with bulbil development

A comprehensive analysis of factors affecting plant bulbil development shows that hormones and glucose metabolism are the two main core modules of plant bulbil development, which involves a large number of gene regulatory networks. Revealing the regulatory relationship between these genes will provide basic clues and new perspectives for the subsequent study of bulbil.

### 5.1 Gene expression networks that regulate the hormones

Combining what scientists know about how cells and tissues develop bulbils, they’ve found that plant bulbils are caused by controlling gene expression in plant meristematic tissue cells, which leads to plant cells being totipotent (Guzman-Lopez et al., 2016).

**Figure 6:**
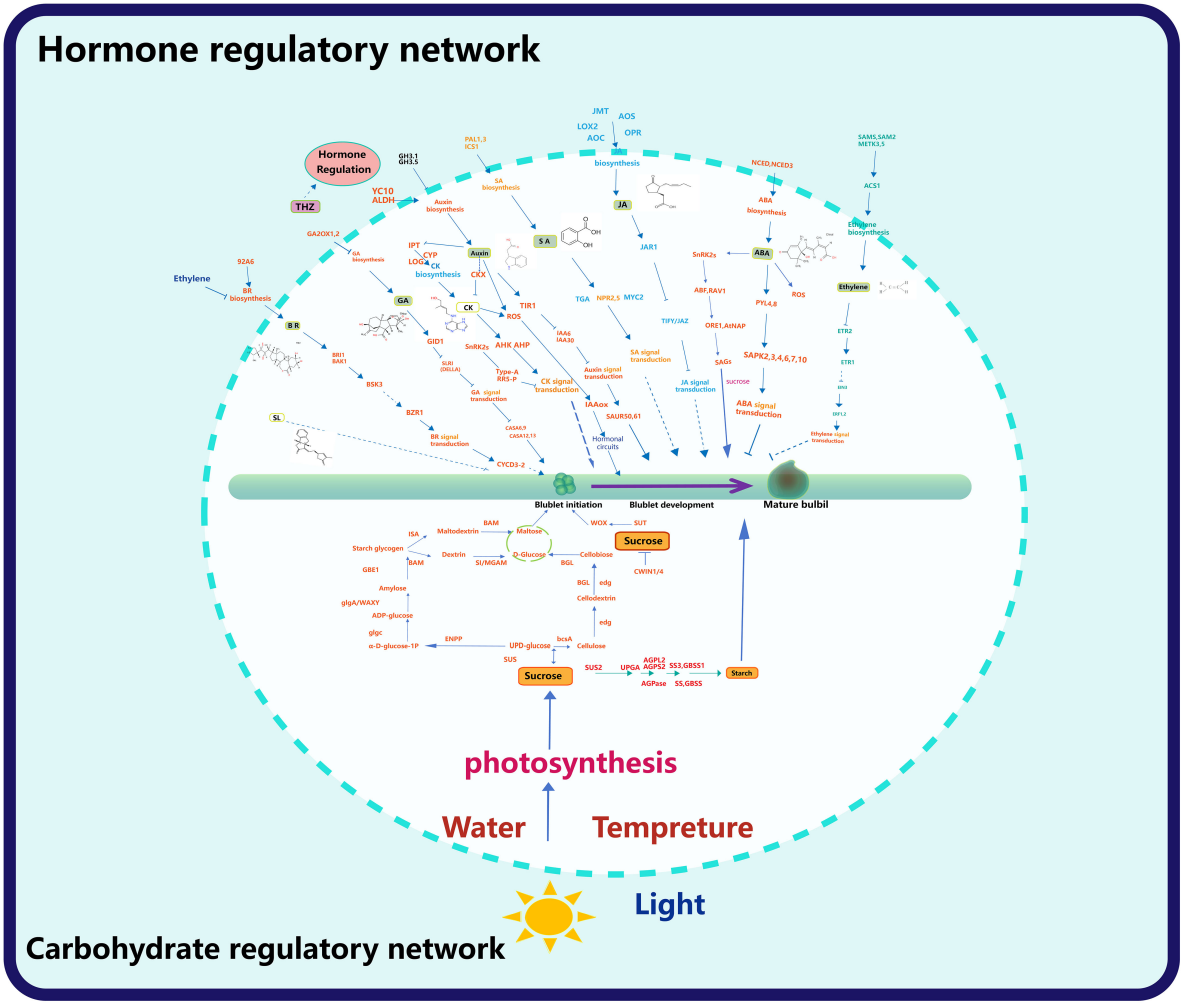
Hormone and sugar gene regulatory network. The upper part of the circle shows the regulatory network of bulbil formation and later development by the hormone, and the lower part of the circle shows the regulatory network of the carbohydrate, light, water and temperature on bulbil formation and later development.

Upregulation of *YUC10* and *aldehyde dehydrogenase* (*ALDH*) promotes auxin synthesis (Xu et al., 2020 ; Li et al., 2022). IAA plays an important role in the initiation of bulbil, and the concentration of IAA is upregulated at this stage. GH3 can regulate the combination of IAA and amino acids, leading to a decrease in IAA concentration, which leads to the failure of IAA regulation on bulbil initiation (Yang et al., 2017). Auxin signal transduction is inhibited by transport inhibitor response 1 (TIR1) and positively regulated by auxin response factor (ARF), and ARF is regulated by auxin, which may be a feedback loop (Chapman and Estelle, 2009; Xu et al., 2020). The small auxin up RNA *(SAUR)* gene can regulate auxin synthesis and transportation and affect auxin signaling. In *Lycoris radiata*, some *SAUR50/61* genes are up-regulated, at which time bulbil initiation occurs (Xu et al., 2020). In *Pinellia ternata*, the expression of the *SAUR* gene was downregulated after BR treatment, and the bulbil was developed (Guo et al., 2021).The *PIN-FORMED (PIN)* gene family and *like auxin1 (LAX)* family of auxin influx carriers are two major gene families that control the auxin distribution (Chapman and Estelle, 2009; Chen et al., 2023a). Upregulation of *PIN, Auxin/IAA, ARF, ALDH* during up- bulbil initiation in behead lily (Li et al., 2022). However, *LAX* was up-regulated at the bulbil initiation stage (Xu et al., 2020). It was previously discussed that *IAA* plays a dual role in bulbil initiation, and the upregulated expression of *LAX* may be in preparation for changing the distribution of IAA, (**Figure 6**).

In the process of bulbil initiation, the content of CK and auxin had the opposite trend, but both played an important role (Xu et al., 2020). In this process, the key genes of CK biosynthesis, *IPT5* (encoding *adenosine monopentenyl transferase*), CK activating enzymes *LOG1* and *LOG5* (encoding *cytokinin nucleoside 5 ‘- monophosphate phosphate hydrolase*), and *CYP735A1* (encoding *a cytokine hydrolase that catalyses the biosynthesis of trans zeatin*), were up-regulated, which promoted the synthesis of CK. However, the content of CK did decline at this stage because the gene *cytokinin dehydrogenase (CKX)* related to CK degradation is significantly up-regulated (Xu et al., 2020; Li et al., 2022). This is not contradictory because the time of transcription from the gene is ahead of the time of CK synthesis, while CK degradation proceeds simultaneously. *Histidine kinase (AHK)* is associated with the transduction of CK, and *AHK2* is significantly upregulated during bulbil initiation (Liang et al., 2023) (**Figure 6**).

*GA2OX* plays a key role in GA metabolism because it will synthesise GA2-oxidase, catalysing the inactivation of GA or its precursors for improved plant architecture and reduced biomass recalcitrance (Wuddineh et al., 2015). GID is a family of F-BOX proteins, and the *GID* can encode a receptor for GA (Xu et al., 2020). While the DELLA protein is a signaling inhibitor of the GA encoded by *SLR1*, whose expression by *GID* leads to the degradation of *SLR1* (Ueguchi-Tanaka et al., 2008). *GID* was downregulated after the IAA application, while *DELLA* showed the opposite trend (Xu et al., 2020; Li et al., 2022). Later, *GA-stimulated Arabidopsis (GASA)*, transduced the GA signal to promote the initiation of the bulbil (Xu et al., 2020), (**Figure 6**).

In the regulatory network of ABA, *9-cis-epoxycarotenoid dioxygenase (NCED)*, a rate-limiting enzyme in ABA synthesis, is significantly downregulated during lily bulbil initiation (Xu et al., 2020; Li et al., 2022).*PYL 8* and *serine/threonine-protein kinase (SAPK)*, which are involved in ABA signaling, were also downregulated in apical lily removal (Li et al., 2022). *PYL* was also downregulated during bulbil initiation in *Pinellia ternata*, and *PYL* was significantly upregulated after treatment with PCX, an inhibitor of BR (Guo et al., 2021). This suggests that suppressed ABA hormone signaling contributes to bulbil initiation, (**Figure 6**).

In the regulatory network of BR, the *P450* and *92A6* genes involved in BR synthesis are significantly upregulated during bulbil initiation (Xu et al., 2020). The downstream genes of BR, *BRASSINOSTEROID INSENSITIVE 1-ASSOCIATED KINASE 1 (BAK 1)*, *BR signaling kinase 3 (BSK 3)*, and *BRASSINAZOLE RESISTANT 1 (BZR 1)* and *BR signal transduction gene CYCD 3-2* were all upregulated (Xu et al., 2020; Guo et al., 2021).

In the regulatory network of ethylene regulating bulbil initiation or development, the genes related to ethylene biosynthesis and metabolism (*SAMS/METK*) and *1-aminocyclopropane-1-carboxylic acid synthase (ACS1)* were down regulated. Corresponding to these are genes related to ethylene signal transduction, including *ethylene receptor 2* (*ETR2*), *Ethylene intrinsic 3 (EIN3)*, and *ethylene responsive transcription factor 2 (ERF)*, *ETR*, *ACO* were significantly downregulated. In addition, a negative regulator of ethylene signaling *constructive triple response 1 (Ctr1)* was unregulated (Xu et al., 2020; Li et al., 2022), (**Figure 6**).

In the regulatory network of JA regulating bulbil initiation or development, JA synthesis and JA-related genes JA-methyltransferase (JA) and JA signaling *JA-amido synthetase* (*JAR 1*), while the *TIFY* family of genes that hinder JA signaling pathway is downregulated (Xu et al., 2020). However, after lily decapitation, both low and high concentrations of IAA induced the wound bulbil initiation, and the genes *LOX 2* associated with JA synthesis, *AOS, AOC, OPR* and genes associated with JA signaling, *MYC 2* were significantly upregulated, while *JMT* was significantly downregulated. At the lower end of the wound, the vast majority of gene expression associated with JA was opposite to the wound end, and treatment with IAA caused a decrease in bulbil initiation at the lower stem (Li et al., 2022). This implies that the crosstalk of IAA with JA can regulate bulbil initiation, with increased IAA, and increased JA promoting bulbil initiation, (**Figure 6**).

In the regulatory network of SA regulating bulbil initiation or development, the enzymes *isochorismate synthase 1 (ICS 1)* and *phenylalanine ammonia-lyase (PAL)* related to SA synthesis were upregulated, and the genes related to signaling *NPR* and *TGA* were also significantly upregulated (Xu et al., 2020; Li et al., 2022). *PAL* and *TGA* were mostly downregulated at the lower end of the lily wound, while bulbil initiation decreased after the use of high concentrations of IAA (Li et al., 2022). This also implies that SA upregulation and IAA upregulation promote bulbil initiation, (**Figure 6**).

In the regulatory network of SL regulating bulbil initiation or development, *carotenoid cleavage dioxygenase 8 (CCD 8)* is the synthetic key gene of SL, and its expression is inhibited after the lily. *Decreased apical dominance 2 (DAD 2)* and *dwarf 14 (D14)* is involved in the signal transduction of SL, and its high expression may be responsible for the inhibition of bulbil initiation in the lower part of decapitated plants (Li et al., 2022), (**Figure 6**).

### 5.2 Gene regulatory networks that regulate sugars

It has been discussed before that the role of sugar in the process of bulbil development is mainly that soluble sugar promotes the initiation of bulbil and starch promotes the late development of bulbil. This involves a very complex mechanism of gene regulation. However, clarifying these regulatory relationships is an important clue for bulbil initiation and development.

Combining the basic knowledge of cell organisation and bulbil development, some scholars attribute the occurrence of plant bulbils to regulating the gene expression of plant meristem cells to stimulate the dry expression of plant cells (Yang, 2018). The analysis of the expression pattern of llarr1 showed that the expression level of *ARR1* was high in the period of bulbil preparation of *Lilium lancifolium*, and it could be expressed in the axil of leaves, indicating that *ARR1* may be involved in the occurrence and development of bulbil of *Lilium lancifolium*, and play a role in this process (Chen et al., 2023b). Overexpression of *LBD 18* in *Lilium lancifolium* can promote bulbil initiation, while bulbil initiation after silencing of *LBD18* is significantly inhibited, indicating that *LBD18* plays a positive regulatory role in bulbil initiation in *Lilium lancifolium*. In the study of sucrose metabolism-related genes in *Lilium lancifolium*, it was found that the activity of the SUSY decomposition direction was significantly increased and the activity of the SUSY synthesis direction was significantly decreased during the initiation of bulbil primordium, which accelerated sucrose degradation, reduced sucrose concentration in leaf axils, enhanced sink strength, and promoted sucrose input and starch synthesis. The expression of SUSY increased slowly in the early stage of bulbil initiation, while it increased sharply in the late stage from axillary uplift to bulbil initiation, and reached the maximum at the time of bulbil structure initiation (Chen et al., 2023b). Eleven members of *PEBP* gene family were identified in *Dioscorea opposita Thunb*. Among them, the *DPTFL1* gene was only expressed in the axils of leaves with stem bulbils in *Tieguan Dioscorea opposita Thunb*. The stem bulbils developed, and the expression of the *DPTFL1* gene gradually increased, reaching its highest level at the mature stage (Sun et al., 2021). In *Lycoris radiata*, seven genes encoding sucrose synthase (SUS) and three *UDP glucose pyrophosphorylase (UGPase)* were up-regulated during the initiation of bulbils, but these genes were downregulated during the late development of bulbils (Xu et al., 2020). In this process, the genes related to starch synthesis (*SS2, GBSS 1, AGPS 2, AGPL 2*, *granule-bound starch synthase (GBSS), and ADP glucose pyrophosphorylase (AGPase)* small and large subunits were downregulated in the bulbil initiation stage and up-regulated in the later stage of bulbil development. This suggests that sucrose synthesis is required during the initiation stage of bulbil and starch synthesis during later developmental stages. Correspondingly, genes, during bulbil initiation, such as *invertase (INV)*, *fructokinase (FRK)*, and *phosphogs invertase (PGM)*, the genes were not up-regulated, which proved that sucrose synthesis plays an important role in bulbil initiation (Li et al., 2022). In *Asiatic hybrid lily*, CPPU and 2-iP treatment promoted bulbil initiation, during which the *sucrose synthase SUS 1/2* was upregulated and *sucrose cell wall invertase (CWIN 1/4)* expression was inhibited (Liang et al., 2023). In *Pinellia ternata*, *enzymes sucrose synthase (SUS)*, *ectonucleotide pyrophosphatase/phosphodiesterase family member 1/3 (ENPP)*, *glucose-1-phosphate adenylyltransferase (glgc)*, *starch synthase (glgA)*, *granule-bound starch synthase (WAXY)*, *1,4-alphaglucan branching enzyme (GBE1), beta-amylase(BAM), sucrase-isomaltase (SI), maltase-glucoamylase (MGAM), isoamylase(ISA), cellulose synthase (bcsA), edg*, the activity of and BGL is closely associated with the metabolism of starch and sucrose. The corresponding upregulation of genes encoding these enzymes is closely related to their activity. Upregulation of *BGL* and *edg*, while *BAM* and *ISA* are downregulated (Guo et al., 2021). This result is similar to that of the BR-induced increased cell wall glucose in switchgrass cell suspension cultures (Rao et al., 2017). Sucrose can also induce the expression of *WUSCHEL-related homeobox 11/9 (WOX 11/9)* (He et al., 2022), *LlRR 1/2/9/10/11/12* (He et al., 2021, 2022), *SUS*, and *SUCROSE TRANSPORTER 2 (SUT 2)*, and *WOX 11* is a transcription factor that can promote the regeneration of plant organs (Wan et al., 2023), *SUT 2* plays an important role in the transport process of sucrose (Zhang et al., 2016). In addition to the two core modules of hormone and sugar, there are many unidentified genes involved in the regulation of bulbil development, for instance: *GFLO* (Li et al., 2014; Xu et al., 2020; Zhang et al., 2023a), *KNOX1/2/6* (Abraham-Juarez et al., 2010; Zhang et al., 2013; Peng, 2016), *MADS1/2/4/6/7* (Peng, 2016; Wu et al., 2019), *LFY* (Peng, 2016), *GRAS* (Peng, 2016), *LOB* (Peng, 2016), F-box (Peng, 2016), *PteMFT* (Liu, 2018), *PEBP* (Liu et al., 2018), *ARF* (Li et al., 2023b), *GFLO* (Wang et al., 2004), *CCD8* (Sun et al., 2011), *IPT1* (He et al., 2020), *CKX4* (He et al., 2020), *LOG* (He et al., 2020), *PIN* (Abraham et al., 2015). There are also a large number of transcription factors such as *bHLH* (Wu et al., 2020; Li et al., 2022), *WRKYs* (Li et al., 2022), *TCPs* (Li et al., 2022), *MYBs* (Wu et al., 2020; Li et al., 2022), *NACs* (Li et al., 2022), *E2F* (Wu et al., 2020), *C2H2* (Li et al., 2022), *bZIP* (Li et al., 2022), etc. These transcription factors are related to sugar and hormone signals (Li et al., 2022), and may play an important role in the initiation of bulbils, however, more evidence is needed to prove it. Between these molecules will occur, making the regulation mechanism of bulbil initiation more complex, (**Figure 6**).

To sum up, research on genes related to plant bulbils mainly focuses on the past decade, mainly due to the rapid development of gene sequencing technology and the continuous reduction of costs. However, many genes related to bulbil development in bulbil-plants have not been reported. Among the reported genes, the research mainly revolves around two major focal points: genes related to hormone regulation and genes related to sucrose regulation. Most of these genes are involved in differential expression screening, gene cloning, bioinformatics analysis, and relative expression level changes, and progress in research involving specific molecular mechanisms is very slow. Moreover, while studying the expression of these genes, there is a disconnection in the description of plant systemic associations, such as how metabolites in other parts of the plant affect bulbil development by influencing gene control.

## 6 The phenomenon of multiple bulbils

**Figure 7:**
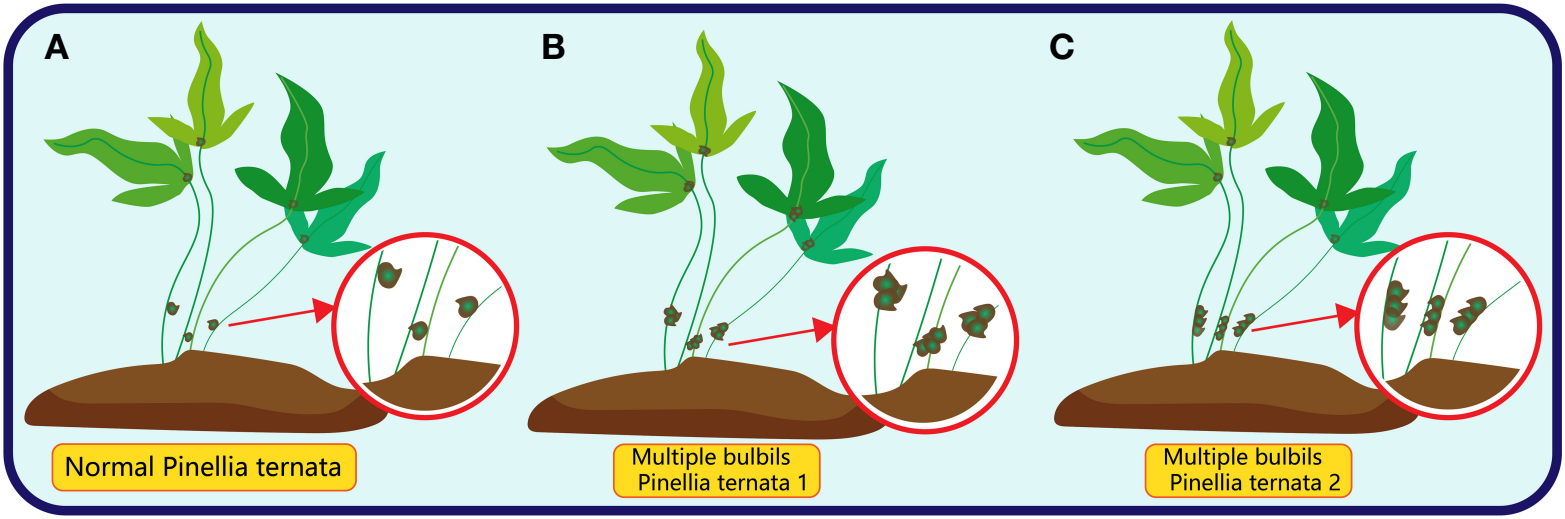
The red circle in **(A)** represents the normal bulbil (only one) of *Pinellia ternata*; The red circle in **(B)** represents the multiple bulbils of *Pinellia ternata* in an irregular order; The red circle in **(C)** represents the multiple bulbils of *Pinellia ternata* arranged in a line.

Multiple bulbils, characterised by the initiation of more than one bulbils on a single plant body, while infrequent, have been observed in certain plant species (Tian et al., 2021; Yuan, 2023; Li et al., 2023b). In *Pinellia ternata*, there are two common types of multiple bulbil: the first type is more than two bulbil at the location of bulbil, which has no specific arrangement of traits; the other is more than two bulbil near the location of bulbil, and the bulbil are arranged neatly along the stalk (**Figure 7**).

Multiple bulbils initiations can be caused by various factors, including: (a) Abnormal hormone balance: If certain hormone levels within the plant become unusually elevated, it may lead to the initiation of multiple hormones (Zhang, 2019; Tian et al., 2021). (b) Genetic mutations: Some genetic mutations can result in plants producing multiple bulbils, for example, in *garlic*, the multiple bulbil type belongs to the first type, and the analysis of the isoenzyme genotypes of garlic in different regions found that garlic in Central Asia has more and smaller bulbils, and these differences are likely due to genetic variation in the isoenzyme (Hirata et al., 2016). (c) External treatments: For instance, the application of high concentrations of plant hormones or specific environmental stimuli may induce the development of multiple bulbils (Jiang and Asami, 2018), we have previously analysed the subtle role of hormones in the initiation of bulbils, but the specific mechanism of the phenomenon of multiple bulbils is still unclear. However, we speculate that one reason for the initiation of multiple bulbils may be that the plant breaks the balance after being stimulated by external substances such as CPPU and 2-ip (cleavage hormone analogues), which stimulate the division of the meristem at the bottom of the original bulbils to form multiple bulbils connected at the base. (d) Stress response: certain environmental stresses, such as water stress: Water stress can cause the rapid response of plant ABA hormone, has elaborated that ABA has an important influence on the development of bulbil, and it should also be noted that ABA can induce the production of ethylene induced plant leaf senescence induced SAGs gene expression, and SAGs can transport nutrients such as sugar to the lower nutrient storage organs, and bulbil is an important nutritional organ of plants (Xu et al., 2020), Meanwhile, water stress causes an increase in ROS (Li et al., 2023c), while ROS are the signaling molecules for cell division (Lee et al., 2017; Wang et al., 2021), Then this has the conditions for the bulbil initiation, especially under the repeated regulation state of the water stress and the normal water supply. Another reason is insufficient light, where KARS (a small molecule isolated from tobacco that promotes germination) promotes seed germination through the biosynthesis of transcriptionally activated GA (Xu et al., 2023), and the up-regulated expression of many members of GA-stimulated Arabidopsis (GASA) may play an important role in the initiation of the bulbil (Xu et al., 2020). In summary, the initiation of bulbil is a complex process involving various cellular and molecular mechanism. While the phenomenon of multiple bulb initiation is not common, it can occur in specific situations, providing plants with more opportunities for asexual reproduction.

## 7 Medicinal value of bulbils

*Lilium lancifolium Thunb*. bulbils have a lower crude fat content and a higher crude fibre content compared to the bulbil, with no significant difference in total sugar, crude protein, or ash content. Elemental analysis shows that both lily tubers and bulbil are rich in various nutrients, especially potassium. The amino acid composition is similar in both, with the highest content of proline. Bulbils are high in total phenols, flavonoids, and saponins, but low in alkaloids (Zhu et al., 2016). In *Pinellia ternata*, the medicinal component content of bulbils is similar to that of tubers of the same size, with alkaloids around 0.04%, polysaccharides between 3.12% to 4.33%, and guanosine between 0.0078% to 0.013% (Li, 2014). After BR treatment, the total flavonoid content in *Pinellia* bulbils is even higher than in the tubers (Guo et al., 2022). An analysis of the medicinal and nutritional components of *Polygonum viviparum* bulbils shows they are rich in protein (9.6%), starch (24%), crude fibre (7.3%), and crude fat (15%). The total bioflavonoids in the bulbils, which make up 3.6% of their dry weight, far exceed the content in plants like sea buckthorn and ginkgo leaves, and are non-toxic. Additionally, the high tannin content (0.24%) in bulbils is effective in treating stomach diseases. The bulbils of *Polygonum viviparum* also contain various minerals and amino acids, including eight essential amino acids: Val, Leu, Ile, Lys, Thr, Met, Phe, Try, and two semi-essential amino acids, Arg and His, playing important roles in metabolism (Weng, 2011). They also contain a significant amount of unsaturated fatty acids (Hu et al., 2008). These medicinal components are important in treating epilepsy (Deng et al., 2020), reducing acute lung injury caused by lipopolysaccharides (Yang et al., 2023b), antioxidation (Ye et al., 2023), and anticancer (Zhao et al., 2022). However, these benefits are not limited to the content level of medicinal components; some bulbils’ mixed extracts have shown even greater medicinal value. For example, a study on the methanol extract of *Dioscorea opposita* bulbils showed that it inhibits TNF-receptor-induced adhesion molecule expression by suppressing the *MAPK*, *AKT*, and *NF-KKB* signaling pathways, thereby reducing inflammation in atherosclerotic lesions (Choi et al., 2012). Extracts from the bulbils of *Dioscorea bulbifera L*. are used to treat Alzheimer’s type dementia (Sadhu et al., 2014). In conclusion, plant bulbils contain a wealth of medicinal components, some even more so than the traditionally used tuber parts. Moreover, harvesting bulbils does not lead to plant death, thus greatly preserving herbal medicine resources and increasing the yield of other herbal parts. This suggests that utilizing bulbils in the development of traditional herbal medicines is highly promising.

## 8 Value of bulbils in breeding

The development of bulbils, represents a strategic adaptation employed by specific plant species in response to particular environmental challenges, such as cold, drought, or low light (Walck et al., 2010). Moreover, these bulbils serve as an efficient method of asexual reproduction (Steiner et al., 2012). For instance, certain Allium species showcase the production of bulbil at the tips of their inflorescences (Kim et al., 2006). Traditional cultivation of *Amorphophallus konjac*, *Amorphophallus albus*, and *Pinellia tilizi* has long been plagued by a bacterial affliction known as soft rot disease, significantly jeopardizing their yields (Shu et al., 2021; Li et al., 2023d; Zhang et al., 2023b). However, recent attention has turned towards a specific subset of *Amorphophallus konjac*, known as bulbil *Amorphophallus konjac*, tilizingnd by the growth of aerial bulbil corms along major leaf veins, enhancing their reproductive capabilities (She et al., 2018). This unique growth pattern elevates their reproduction coefficient. Furthermore, researchers have identified soft-rot-resistant varieties of *Amorphophallus konjac*, with the *Maitake Amorphophallus konjac* being a notable example, and these varieties have been widely propagated (Dai et al., 2023). The tubers take years to breed again, and raising the bud is an effective way to quickly obtain lily seeds (Li et al., 2022).

Furthermore, *Pinellia tilizi* holds medicinal value in the bulbil (Feng et al., 2012). The initiation of bulbils in *Pinellia tilizi* primarily occurs towards the end of the growing season, particularly from late summer to early autumn. During this period, plant growth gradually slows down, and nutrients begin to shift from leaves to underground organs, especially the bulbil. As nutrients accumulate, specific parts of the bulbil initiate development as new bulbil shoots. These bulbils can germinate and give rise to new plants in the subsequent growing season. This area of research holds significant practical value for the high-quality production and tilizingn of herbal medicines (Zhang, 2016, 2017).

It’s worth noting that bulbil themselves can generate new bulbil (He et al., 2019). When a bulbils germinates and initiations grow under suitable conditions, it forms its bulbil. As this new bulbil develops, it may also begin producing new bulbil on its surface. In this manner, plant can reproduce through bulbil and continuously expand their population through a continuous process (Luo, 2010). A significant advantage of this reproduction method is its ability to rapidly multiply and spread within a relatively short timeframe. However, the initiation and development of bulbil necessitate adequate nutrients, which means that plants must accumulate sufficient nutrients during the growing season to support the initiation and germination of bulbil. However, this is evidently a matter determined by nature and time. What we need to emphasise is that all plant bulbils play a crucial role in increasing the quantity and quality of their populations. Furthermore, since most bulbils grow on the stems of bulbils plants, they are seldom infected by pathogenic microorganisms. This establishes a solid foundation for tilizing bulbils as high-quality germplasm in the coming years.

Among flowers, flowering plants account for a large proportion, and among these flowering plants, lilies, agave and other common flowers are also important bulbil- plants. Flowering plants have an unparalleled diversity mechanism to achieve sexual reproduction and asexual reproduction, which are usually carried out at the same time (Barrett, 2015). The genetic diversity brought by sexual reproduction and the rapid increase in the number of plant populations, such as garlic (with many bulbils) (Hirata et al., 2016), are not only the source of new varieties but also the disaster of high-quality varieties for flowers and Chinese herbal medicine plants, because people pay attention to the biological characteristics of good varieties, such as colour, character, the number of flowers, flower cycle, medicinal ingredients, etc., are likely to greatly reduce the stability of these traits after sexual reproduction, genetic stability is also the firm basic demand of new varieties (Cowling et al., 2023). In addition, the seed cycle produced by sexual reproduction is too long and the reproductive ability is weak, which is extremely unfavourable to the early listing of flowers, Chinese herbal medicine, etc. In the vegetative reproduction of plants, the role of bulbil is particularly noticeable, especially when compared with the traditional seed based sexual reproduction. This comparison not only reveals the diversity of plants’ adaptation to environmental pressure and diffusion strategies, but also emphasizes the unique advantages of asexual propagation of bulbils and its significance for plant biology research. Specifically, the process of bulbil forming new individuals does not involve seed formation or sowing, which brings two major advantages: (1) it is highly consistent with the parents’ heredity: because it is directly propagated from a single parent, the offspring maintain almost identical genetic characteristics, which is very important to maintain the characteristics of a specific variety, especially in flowers, the economic loss caused by the poor genetic stability is huge. (2) Rapid diffusion of plant population: corms can quickly form new individuals and accelerate the diffusion of plants, especially under favourable environmental conditions. Therefore, plant bulbil is a very potential provenance in plants that need stable traits or accurate breeding according to stable traits, such as flowers.

## 9 Construction of a plant tissue culture induction system for bulbils

The promising prospects of bulbils in medicine and breeding have attracted significant research interest, with plant tissue culture being the most widely used technique (Loyola-Vargas and Ochoa-Alejo, 2018; Ji et al., 2023). This method allows year-round production with controlled indoor conditions like light, temperature, and humidity. It forms a basic part of increasing bulbil yield and understanding bulbil initiation mechanisms. The bulbils induced in this sterile environment are excellent sources for propagation. For *Lilium longiflorum* (Easter Lily), a propagation system for bulbils was established as early as 1998. Dormant bulbil apices were put on 1/2 MS medium and then moved the new growth with nodes to 1/2 MS medium that had 1 mmol/L 6-BA in it. Bulbils were obtained after 30 days (Nhut, 1998). Subsequently, numerous induction systems for lily bulbils were established (Li et al., 2019, 2022). Inspired by lily research, other bulbil plants have developed tissue culture systems. For instance, in 2007, a successful culture system for *Pinellia ternata* bulbils were established using MS medium with 6-BA 1 mg/L, IAA 0.5 mg/L, 3% sucrose, and varying concentrations of calcium (Chang, 2007). A long time ago, a tissue culture system for *Curculigo orchioides* bulbils was developed using B5 medium with 200 mg/L KNO3, 50 mg/L (NH4)2SO4, 2.2 μmol/L 6-BA, 0.11 mmol/L adenine, 1 μmol/L IBA, and 250 mg/L PVP. Later, the medium was optimised to MS with 1 mg/L BA and 0.1 mg/L morphactin, which increased the induction rate (Nema et al., 2008) . For *Dioscorea opposita* bulbils, the culture medium is MS with 4.0 mg/L 6-BA, 1.0 mg/L IBA, 6%~9% sucrose, 0.5% activated charcoal, and 0.7% agar (Peng, 2005). For Chinese *Dioscorea opposita* bulbils, the medium is MS with 1.5 mg·L^-1^ 6-BA, 0.2 mg·L^-1^ NAA, and varying sucrose concentrations; 1%~3% sucrose is beneficial for bulbil induction (Han et al., 2013).

Analysis reveals significant differences in culture medium formulations for different plants. Many plant species with potential bulbil tissue culture systems remain unreported. The establishment of these systems involves complex engineering, with breakthroughs likely in the establishment of basic media, the correct selection of hormones, and the appropriate combination of sucrose concentrations. While many species’s bulbil culture systems are yet to be established, the reported systems have laid a good foundation for *ex vitro* induction of bulbils. Whoever combines these systems with more advanced equipment or techniques to expand bulbil production will significantly alleviate the shortage of high-quality germplasm resources, opening a new chapter in the utilisation of bulbil resources.

## 10 The temporary immersion bioreactor system is a very promising device for the large-scale breeding of bulbils

**Figure 8:**
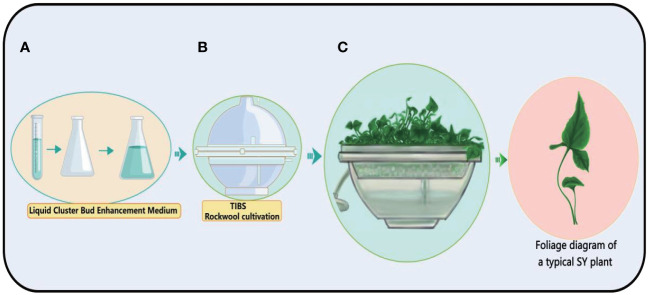
Schematic diagram of the process of TIBS culture of *Pinellia ternata.* The plant cultivation process of TIBS is very simple. First, the culture medium **(A)** required by the plant is prepared, and then placed in TIBS for sterilization **(B)**, and the sterile plant is placed in TIBS, and the sterile plant is continuously expanded **(C)**.

The Temporary Immersion Bioreactor System (TIBS) is a carefully designed machine that creates the best environment for cells or tissues to grow (Le et al., 2021; Uma et al., 2021). In the field of plant biology, TIBS occupies a prominent position and is primarily focused on amplifying the production of secondary plant metabolites, a ground-breaking application (Mirzabe et al., 2022). This clever system allows precise control over a wide range of outside factors, such as light, temperature, and the availability of nutrients (Deng et al., 2021; Qian et al., 2024). This makes it possible for plants to grow faster and make more secondary metabolites. Bioreactors assume a pivotal role in this endeavour by furnishing a meticulously controlled environment for plant cells, thereby facilitating the optimisation of conditions conducive to the genesis of bulbil. Obtaining the seedlings of bulbil plants is an important step in obtaining bulbils. The BioF Temporary Immersion Bioreactor System (TIBS) is a novel TIBS device that is widely used for rapid propagation of bulbils plants such as *Pinellia ternata*, *Lilium sulphureum*, and *Amorphophallus konjac.* In fact, a large number of bulbil plant resources have not been developed, and the BioF bioreactor has great potential for tapping these resources. We have observed in production that bulbil can be directly obtained by using a BioF bioreactor, which greatly shortens the time of obtaining bulbil. For a visual representation of this innovative system, (See **Figure 8**).

We illustrate the value of TIBS applications in detail with a few specific Bulbils plant examples. *Pinellia ternata, Lilium sulphureum*, and *Amorphophallus konjac* all grow in very different ways because their biology, life cycles, and environmental needs are all very different. *Pinellia ternata* is an herbaceous plant native to East Asia, thriving in shaded and moist environments often found in woodlands and along stream banks. This low-growing, herbaceous perennial exhibits a distinctive growth form, characterised by a single trifoliate leaf and a unique inflorescence. Its reproductive strategy encompasses both seeds and bulbils, which are produced in the leaf axils. Typically, *Pinellia ternata* experiences active growth during the spring and summer seasons, transitioning into a dormant state during the winter months. This plant’s adaptation to its habitat and seasonal growth patterns contributes to its resilience and ecological significance in its native regions. *Pinellia ternata* is renowned for its medicinal attributes, housing a diverse spectrum of bioactive compounds (Lu et al., 2020). Notably, when cultivated within a TIBS, *Pinellia ternata* experiences accelerated growth and an augmented accumulation of its bioactive constituents (Wang D. et al., 2023). The development of bulbils in this plant is a multipart process, encompassing initiation, growth, differentiation, and maturation stages. This intricate growth and bioactive compound accumulation process underlines the plant’s significance in medicinal applications and the potential for enhanced cultivation methods through innovative bioreactor technology.

*Lilium sulphureum*, the exquisite trumpet-shaped flowers of this bulbous perennial plant, which come in a variety of colours from yellow to orange, are what set it apart. The primary mode of reproduction for *Lilium sulphureum* is through bulbil or tubers, although it also has the capacity to produce seeds. Typically, its growth season unfolds during the spring and early summer, when it graces the surroundings with its vibrant and captivating blooms. This plant’s unique habitat preferences, striking flowers, and reproductive strategies contribute to its ecological and horticultural significance. *Lilium sulphureum is* renowned not only for its edible and ornamental attributes but also for its substantial medicinal value (Liang et al., 2022). When cultivated within a TIBS environment, *Lilium sulphureum* exhibits accelerated propagation and enhanced accumulation of valuable bioactive compounds; this process is quite similar to *Dendrobium cariniferum Rchb. F* culture in TIBS/BioF (Lin et al., 2020). This multifaceted utility underscores the plant’s significance in both culinary and therapeutic applications, with the potential for advanced cultivation techniques in TIBS systems contributing to its broader utilisation.

*Amorphophallus konjac* originates from Southeast Asia and flourishes in tropical and subtropical regions. This herbaceous perennial assumes a distinct growth form, characterised by its substantial underground corm, from which a solitary, umbrella-like leaf emerges. Its primary mode of reproduction relies on corms, which have the capability to generate offsets, although seed production is less frequent. Typically, *Amorphophallus konjac* experiences active growth during the warmer months and enters a dormant phase throughout the winter. This unique combination of habitat preference, growth structure, reproductive strategy, and seasonal growth patterns contributes to the plant’s resilience and adaptability to its native environments. *Amorphophallus konjac*, celebrated for its glucan-rich roots that serve as vital raw materials for diverse food and chemical products, offers substantial versatility and utility. Through cultivation within TIBS, the granular callus of Amorphophallus konjac was used (Wu et al., 2008) to study the propagation of the callus in bioreactor. The results showed that the diameter of Amorphophallus konjac callus increased from 0.3cm to 0.8cm after 12 days of cultivation in a bioreactor with an inoculation amount of 10 g/6L, ventilation rate of 60 L/h and a stirring speed of 100r/min (Wu et al., 2008). This process enhancement augments the plant’s economic and industrial value, emphasising its potential in various applications (**Figure 8**). These plants exhibit diverse growth characteristics, adapting to their respective habitats and climates. *Pinellia ternata* is known for its unique leaf structure and ability to produce bulbils; *Lilium sulphureum* for its striking flowers; and *Amorphophallus konjac* for its large underground corm and unique leaf structure. Understanding their growth patterns and requirements is essential for their successful cultivation and management in TIBS.

## 11 Terahertz offers great potential for developmental research and the production of plant bulbils

Terahertz (THz) technology offers a novel avenue for investigating plant biology. Recent research has unveiled specific responses in plants like *Pinellia ternata* (Wang D. et al., 2023). THz is intimately linked to the physiological and biochemical processes in plants and provides a potent tool for delving deeper into plant growth, development, and responses to environmental challenges (Li, 2022). Terahertz waves occupy a unique position in the electromagnetic spectrum, falling between microwaves and infrared radiation with wavelengths ranging from about 0.1 to 1 mm (Chen et al., 2023c; Zhang et al., 2023c). What makes THz technology particularly intriguing in the fields of biology and medicine is its non-invasive and radiation-free capacity to detect molecular and cellular changes (Tan et al., 2019; Zhou et al., 2019). It excels at probing the structure and dynamics of molecules, including phenomena such as protein folding and DNA damage (Wang et al., 2022; Yin et al., 2024). In plant science, THz waves hold promise for exploring their impact on plant growth, development, and stress responses (Gente and Koch, 2015). Theoretically, THz waves may influence water molecules within plant cells, given their heightened sensitivity to water absorption (Zhao et al., 2020). This interaction could potentially alter cell permeability and signalling, subsequently affecting plant growth and development (Wolkers et al., 2019). The regulation of bulbil in plants by THz waves might involve molecular mechanisms and pathways. THz waves may disrupt signalling molecules within plant cells, such as calcium ions and reactive oxygen species (ROS), influencing cell growth and differentiation (Sies and Jones, 2020). THz waves could interfere with the synthesis and transmission of plant hormones, impacting the initiation of bulbils (Fedorov and Tzortzakis, 2020). THz waves might lead to the up- or down-regulation of specific genes, consequently influencing cell behaviour and fate (Cheon et al., 2023). THz waves may modulate the permeability of cell membranes, potentially affecting the uptake of water and nutrients by plant cells (Cherkasova et al., 2021). This research opens up exciting possibilities for understanding the intricate interplay between terahertz waves and plant biology, shedding light on how this technology can contribute to the advancement of plant science. At the same time, new methods are provided for *Pinellia ternata* bulbils research and production.

## 12 Conclusion and future prospects

This article carefully goes over the study of bulbils, including bulbil-plants, their traits, how to tell when they are fully grown, how cells and tissues work during development, what factors affect development, research on genes related to development, the phenomenon of multiple bulbils, their use in medicine and breeding, how to make plant tissue culture induction systems, how to use temporary immersion bioreactors for large-scale production, and the potential. While progress has been made in bulbil research, several unresolved issues remain, mainly in the following areas:

(1) Limited Initiation on Bulbil-Containing Plant Resources: More extensive plant resource surveys are required. This lack of initiation might be due to the underestimation of bulbils by most researchers. Common citizens can contribute significantly to this data collection, with apps like Xingse (2017) and Baidu allowing users to identify known plants and upload images of unknown ones for identification.
(2) Need for Broader Research on Cellular and Tissue Structures Affecting Bulbil Development: Current research primarily focuses on economically valuable plants and examines the cellular and tissue structures of the bulbil sites. However, upstream and downstream tissue structures at the bulbil attachment sites might play a crucial role in determining the occurrence of bulbils. Understanding these relationships can help explain why bulbils often grow at specific sites, like leaf axils or stem bases, and aid in further exploitation of bulbil resources.
(3) Further Research on Bulbil Development Mechanisms: Current research mostly revolves around gene screening, cloning, and expression analysis related to development mechanisms. This approach is insufficient. More in-depth studies could include overexpression or gene knockout experiments, coupled with transcriptomics to identify changes in other genes. Additionally, understanding the input, metabolism within the bulbil, and output of metabolites, which play roles in signalling, nutrient transport, and the synthesis of carbohydrate precursors, is crucial. Metabolomics, closely linked to plant phenotypes, combined with transcriptomics, can yield significant insights into bulbil development.
(4) Potential Applications of Multiple Bulbil Phenomena: The breeding advantage of multiple bulbils is the significant increase in numbers and shortened breeding time. However, compared to single bulbils, multiple bulbils are smaller, which could be addressed by extending cultivation time. The size and cultivation duration are crucial for evaluating breeding potential. Also, the compositional differences between multiple and single bulbils, both in terms of components and their quantities, are critical for developing the medicinal value of different plant bulbils. This component analysis is also essential to assess the feasibility of using multiple bulbils as seed sources in challenging environments like arid, high-light, or saline-alkaline soils.
(5) Refinement and Optimisation of Plant Tissue Culture Systems for Bulbils: The development of bulbil is influenced by many factors. Current tissue culture systems focus on medium formulation, often overlooking the effects of light, temperature, and other factors. A more holistic approach incorporating these elements could lead to superior cultivation methods for bulbils.
(6) Comprehensive application research of TIBS for large-scale expansion of bulbil production: Addressing these issues will provide a vital theoretical foundation for further exploitation of plant bulbils and the advancement of plant science. Technological progress and interdisciplinary collaboration can still yield bulbils on a large scale with current technologies. A promising way to make bulbils is to use tissue culture techniques, temporary immersion bioreactors (TIBS), and terahertz (THz) technology together with factors that affect bulbil development. THz technology, in particular, holds great potential for enhancing our understanding of plant growth, reproduction, and responses to environmental stressors (Long et al., 2022). This innovative combination could lead to significant advancements in agriculture, medicine, and other fields. Plant bioreactors, sophisticated devices designed to create controlled growth environments, allow researchers to precisely manage factors such as nutrients, light, temperature, and other growth conditions, leading to optimised bulbil production. Bulbil represents a form of asexual reproduction in plants, characterised by rapid growth and differentiation of plant tissues under specific conditions. Within a bioreactor, the internal environment can be carefully regulated, including factors like hormone levels, oxygen concentration, and nutrient availability, to efficiently stimulate and support the development of bulbil. These bulbil hold significant value in both plant biology and agricultural practices. First and foremost, bulbils serve as a mechanism for asexual reproduction in plants, allowing specific species to reproduce rapidly while maintaining genetic uniformity. This genetic consistency is vital for preserving crop quality, disease resistance, and other desirable agricultural traits. Additionally, in natural settings, the initiation of bulbil shoots plays a critical role in plant survival and expansion under adverse conditions. In extreme environments where seed survival is uncertain, bulbil sprouts offer a more reliable strategy for survival and reproduction.
By introducing precise quantities of cytokinins and plant hormones, such as BR, into the TIBS, researchers can initiate rapid cell division. As cells continue to divide, bulbils begin to take shape. The controlled environment within the bioreactor ensures the uniform distribution of all necessary factors among plant cells, promoting the healthy and consistent growth of bulbil. Furthermore, the bioreactor ensures a continuous supply of fresh nutrients and efficient waste removal, supporting the optimal development of plant cells. Through vigilant monitoring and regulation of pH, nutrient concentrations, and other critical parameters, researchers can guarantee the rapid and healthy growth of bulbil. The TIBS provides an ideal, controlled setting for cultivating bulbil. This not only expedites the process of asexual reproduction in plants but also ensures that the produced bulbil shoots exhibit high quality and uniformity. This technique boasts a wide array of applications in plant biotechnology and agricultural production. In conclusion, the mechanisms underlying bulbil shoot production in plants such as *Pinellia ternata* involve a complex regulatory network of plant hormones, encompassing intricate interactions among gibberellins, abscisic acid, and growth hormones in stem elongation and bulbil shoot initiation. These processes intricately link to the regulation of specific gene expressions, offering opportunities for in-depth studies and further advancements in plant biology and agriculture.
(7) To find out the common share genetics between bulbils and flowers: It is well known that the same plant to which the bulbil and the flower belong should share the same genetic genes. The bulbil is an important asexual reproductive organ, and the flower is an important sexual reproductive organ. It is a very fascinating direction to study the common genetic mechanism of the two, especially in the common mechanism of gene expression regulation leading to plant directional flower or bulbil differentiation. At present, there is only very limited information about this mechanism, but these information provide important clues for exploring the common genetic mechanism between bulbil and flower. In *Titanotrichum (Gesneriaceae)*, the meristem of flower has three fates: 1) bulbil formation, 2) flower formation, or 3) bracteose promotion. The fast of the meristem is reversible before the division of the first or second meristem. The fate of the primitive is irreversibly determined shortly after the development characteristics of the floor or bulbil pathway appear (Wang and Cronk, 2003). In Agave tequilana, MADS-box transcription factor, which involved in the regulation of floral induction, showed differential expression of these genes in the transition from vegetative growth stage to flowers, as well as in different plant tissues (including apical meristems and inflorescence meristems, floral organs (tepals, anthers, stigma, ovary) and developing bulbils). The specific manifestations are: AtqMADS1 has the highest expression on the stigma in the floral organs. AtqMADS2 The highest expression was observed in the ovary, with a 75-fold change from the meristem of vegetative growth to the “sinking” stage, and about 350-fold from the apical meristem (SAM) to the 1-meter high inflorescence meristem (IM). AtqMADS3 Did not show significant expression magnitude changes between the meristems and floral organs. However, AtqMADS4 expression was highest in all floral organs, with differences in expression levels ranging from 900-fold for teppieces to approximately 3000-fold for anthers. However, AtqMADS1 and AtqMADS2 showed significant changes in expression levels during bud development, with significant expression decreasing during the progression of bulbil development. These results highlight the complex regulation of MADS gene expression, revealing how specific genes are up-or downregulated at different stages of plant development, particularly in the transition from vegetative growth to floral stages, as well as during the development of bulbils (Delgado et al., 2012). These results all point to the genetically shared molecular regulatory mechanism and tissue basis of bulbil and flower, but the current research information is very limited, so it will be a very fascinating field.

## Author contributions

FS: Conceptualization, Data curation, Formal analysis, Methodology, Resources, Software, Writing – original draft. DW: Data curation, Software, Validation, Visualization, Writing – original draft. SS: Formal analysis, Resources, Writing – original draft, Writing – review & editing. LJ: Data curation, Validation, Writing – review & editing. KL: Writing – review & editing. MZ: Resources, Validation, Writing – review & editing. XW: Data curation, Formal analysis, Writing – review & editing. ZY: Data curation, Formal analysis, Writing – review & editing. GC: Conceptualization, Resources, Supervision, Validation, Writing – review & editing. JC: Conceptualization, Funding acquisition, Project administration, Resources, Supervision, Validation, Writing – review & editing.
